# Effects of strain and stocking density on leg health, activity, and use of enrichments in conventional broiler chicken production

**DOI:** 10.1016/j.psj.2024.103993

**Published:** 2024-06-27

**Authors:** M. Guinebretière, L. Warin, J.P. Moysan, B. Méda, F. Mocz, E. Le Bihan-Duval, R. Thomas, A. Keita, S. Mignon-Grasteau

**Affiliations:** ⁎ Epidemiology, Health and Welfare Unit, Ploufragan-Plouzané-Niort Laboratory, French Agency for Food, Environmental and Occupational Health & Safety (ANSES), Ploufragan, France; † Technical Institute for Poultry (ITAVI), 37380 Nouzilly, France; ‡ Avian Experimental Unit, Ploufragan-Plouzané-Niort Laboratory, French Agency for Food, Environmental and Occupational Health & Safety (ANSES), Ploufragan, France; § INRAE, Université de Tours, BOA, 37380 Nouzilly, France

**Keywords:** welfare, behavior, health, genetics, growth rate

## Abstract

Conventional broiler production needs to evolve towards more animal-friendly production systems in order to meet increasing consumer concerns regarding animal welfare. Genetics and stocking density are 2 of the most promising leads to make this change possible. In this study, 6 strains with different growth rates (42–61 g/d) were reared at contrasting densities: 37 kg/m² (**HD**) and 29 kg/m² (**LD**). At the same body weight of 1.80–1.95 kg, we evaluated how growth rate and stocking density influenced broiler behaviors (general activity, interactions with enrichments), broiler health (mortality, leg problems, cleanliness and plumage growth) and litter quality. Density did not affect body weight, mortality or behaviors. For all strains, LD was associated with a lower prevalence of hock burns, a better gait score, and improved litter quality and broiler cleanliness. For the 3 strains most affected by pododermatitis, a lower prevalence was observed in LD than in HD pens. Fewer birds were inactive and more birds were standing and interacting with the enrichments (as proposed in the experiment) as soon as the growth rate was lower than that of the control strain (Ross 308). Others welfare indicators such as gait score, plumage growth improved as well. Litter humidity decreased with growth rate, contributing to better leg conditions and cleaner breasts. The prevalence of hock burns and certain behaviors (i.e., the proportion of birds grooming or walking/running) were not affected by growth rate. The proportion of birds foraging was higher at a lower growth rate. These results suggest that reducing growth rate as a preliminary measure, and reducing density as a supplementary one, would improve conventional broiler welfare.

## INTRODUCTION

In broilers, as in many other animal species, animal welfare in production settings has become a major concern, and research has been increasingly focused on improving our knowledge of the conditions required to improve welfare, including genetics and environmental factors (Vaziri et al., 2022). Indeed, the number of days required by a broiler to achieve the same weight has been divided by 4 since the sixties (Zuidhof et al., 2014) as selection objectives and rearing conditions of conventional broiler chicken have largely focused on improved growth, feed efficiency, and breast yield. Unfortunately, due to the related morphological, physiological or behavioral changes, physical activity and leg health have been impaired (Hartcher and Lum, 2020). Consequently, diverse welfare-related problems are commonly reported in fast-growing broiler chickens. Increased incidences of metabolic disorders (Bessei, 2006), muscle myopathies (Kuttappan et al., 2016), leg problems (Fanatico et al., 2008; Shim et al., 2012), and lameness (Wilhelmsson et al., 2019) are commonly associated with rapid growth. These disorders generally result in suffering for the affected birds. The occurrence of these welfare problems is most often explained by genetics in combination with unfavorable environmental parameters (Bessei, 2006) such as poor litter and air quality, inadequate light programs, and high stocking density. Nevertheless, Dawkins and Layton (2012) hypothesized that the conflict between welfare and productivity can be reduced by using all the available genetic variations concerning growth rate in current strains. Furthermore, high stocking densities limit the expression of several natural behaviors. van der Eijk et al. (2022) showed, for example, that broilers housed at lower stocking densities (24 or 30 kg/m²) displayed more comfort and foraging behaviors than those housed at higher stocking densities (36 or 42 kg/m²). Thus, the use of slower-growing strains and reduced stocking density are considered important welfare improvement levers (Dixon, 2020; Rayner et al., 2020; EFSA AHAW Panel et al., 2023), and are often included in specifications for better welfare systems. For example, the European Chicken Commitment (an initiative of European animal protection organizations) proposes welfare criteria and standards that include a maximum stocking density of 30 kg/m² and slower-growing strains with better welfare outcomes than fast-growing strains (ECC, 2019).

This study is part of a project designed to provide knowledge and solutions to farmers and stakeholders so that most conventional broiler production (representing 85% of French consumption and 74% of French production in 2021 (ITAVI, 2022)) can be developed into a production system with more focus on animal welfare while limiting additional economic costs. This system is intended to be positioned between the current conventional system and the alternative French “**CCP**” product conformity certification system, which complies with private specifications such as an intermediate growth rate, usually lower stocking densities (i.e., 18 chickens/m²), a minimal rearing period of 56 d, and more rarely free-range access. To reach this objective, the welfare of broilers from 6 strains with different growth rates was evaluated. Some were reared at a conventional stocking density of 37 kg/m² and the others at a lower stocking density of 29 kg/m². This study evaluated how growth rate and stocking density influenced behaviors (general activity, interaction with enrichments), mortality and health indicators (leg problems and plumage condition), and litter quality.

## MATERIALS AND METHODS

### Ethics Statement

The housing, management, and experimental procedures all complied with European legislation on the protection of animals used for scientific purposes (EU Directive 2010/63/EU), and were approved by the laboratory's animal welfare structure (No. D-22-745-1).

### Housing and Experimental Scheme

**Figure 1:**
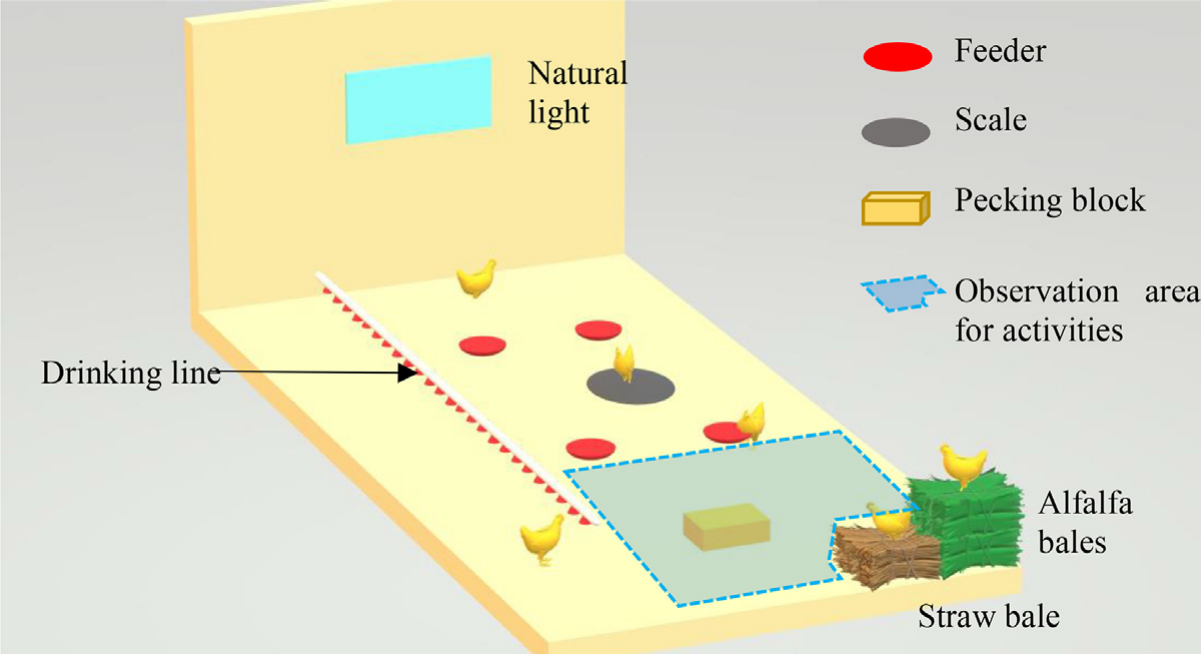
A pen showing the position of the window, drinking line, feeders, and other material: bales, pecking block and scale. The area used to observe activities is indicated in blue.

Six groups of 2,096 one-day-old broilers were provided on the same day to the ANSES Avian Experimental Unit in Ploufragan, France. Each group corresponded to a given strain differing by their estimated average daily gain (**ADG**) for a target weight of 1.80–1.95 kg (ADG provided by the Hubbard and Aviagen breeding companies). They ranged from the current conventional fast-growing strain Ross 308 (61 g/d) to the slower-growing JA 757 strain used in CCP production (42 g/d). The strains with intermediate growth rates tested were Redbro (49 g/d), Rustic Gold (48 g/d), Ranger Classic (47 g/d), and JA 787 (46 g/d). Chicks were placed in 6 identical rooms within the same experimental broiler house (one strain per room). As slaughter was planned at a similar final body weight (around 2 kg), rearing periods differed between strains according to their variable growth rate (Ross 308: 32 d; JA 757: 46 d; other strains: 39 d). Broilers from different strains were placed in different rooms for 2 main reasons: first, this allowed independent control of environmental conditions (such as light and temperature programs) tailored to the needs of each strain. Second, a large number of broilers could be removed at slaughter age without disturbing the remaining birds. In each room, chicks were distributed among 8 identical 15 m² pens: 4 pens with a conventional stocking density of 37 kg/m² (**HD**) and 4 pens with a lower stocking density of 29 kg/m² (**LD**), both for a target BW of 1.80–1.95 kg. Different densities were reached by placing a different number of chickens in HD and LD pens, taking into account a theoretical mortality rate of 4%. Thus, 230 and 300 birds were placed per pen in LD and HD conditions, respectively. Each HD or LD pen (Figure 1) contained one firmly pressed straw bale (15 kg, 30 cm high × 50 cm long × 30 cm wide (Desialys, Paris, France)), 2 firmly pressed alfalfa bales stacked on top of each other (each 20 kg, 30 cm high × 50 cm long × 30 cm wide (Desialys, Paris, France)), a pecking block composed of minerals (oyster shells) and organic inclusions (extruded linseed, brewer's yeast, extruded wheat, and a mixture of aromatic plants) weighing 8 kg and 12 cm high × 19 cm long × 19 cm wide (Pikee Bloc - Wisium, Saint-Nolff, France), and an automatic round hanging scale (8 cm high, 60 cm diameter). The bales and pecking blocks were renewed on a case-by-case basis in the event of marked disintegration.

All the pens were lit by natural daylight from 08:00–19:00 thanks to a window (90 cm × 30 cm), supplemented if needed by artificial light guaranteeing 30 lux for 18 h per day from 6 d of age (from 06:00–23:59). Sawdust litter was provided to birds following conventional management practices (1.1 kg/m²; concrete floor). For the Ross 308 strain, as litter under the drinking line deteriorated, it was removed and replaced with 10 kg of fresh litter on D18 in each pen; moreover, 10 kg of fresh sawdust was added to each pen on D27. All the birds had access to feed and water ad libitum. A commercial feed, formulated to satisfy the requirements of the fastest-growing strain tested (Ross 308), was used to prevent other factors than stocking density and strain from influencing experimental results. A starter diet was given up to D11, a grower diet from D12 to D25, and 2 finisher diets from D26 to D31 (finisher 1), and from D32 to slaughter (finisher 2, for all strains except Ross 308). Feed characteristics were as follows for each diet: starter diet: 2,970 kcal ME/kg, 22.3% CP, 1.05% Ca, 0.50% available *P*; grower diet: 3,035 kcal ME/kg, 20.0% CP, 0.90% Ca, 0.42% available *P*; finisher 1 diet: 3,140 kcal ME/kg, 18.3% CP 0.75% Ca, 0.37% available *P*; finisher 2 diet: 3,210 kcal ME/kg, 16.6% CP, 0.65% Ca, 0.34 % available *P*.

### Data Collection

In order to compare the welfare and health indicators of different strains, the comparisons were based on the following criteria for a similar broiler chicken target weight of 1.80–1.95 kg, reached at different ages depending on the strain. They included mortality rate, litter quality, consumption of bales and blocks, body condition, and broiler behaviors (Table 1).

**Table 1:** Estimated and observed ages at target weight (1.80–1.95 kg), ages chosen for strains and density comparison, and observed age and stocking density at target weight.

Strains	Estimated age at target weight (d)	Ages chosen for comparison between strain × density conditions (d)			Observed age at target weight (d)		Observed density at target weight (kg/m²)	
		Mortality, Litter, Enrichment consumption	Body status	Behavior	HD	LD	HD	LD
**Ross 308**	29-32	31	31	30	29-31	29-30	37.1	28.6
**Redbro**	36-40	39	38	37	37-39	37-39	37.3	28.9
**Rustic Gold**	37-41	39	38	37	38-41	38-41	37.1	28.8
**Ranger Classic**	38-42	39	38	37	39-41	39-41	37.2	28.9
**JA 787**	38-43	39	38	37	39-41	38-41	37.1	29.0
**JA 757**	42-46	46	45	44	44-48	44-47	37.1	29.1

#### Body Weight

A round hanging scale in 2 pens out of 4 per strain × density conditions automatically registered the weight of broilers perched on it. The software calculated a daily mean body weight (**BW**) for the pen. Additionally, 104 randomly selected birds per strain × density conditions (26 per pen, sex balanced) were manually weighed individually on D32.

#### Mortality

The number of dead birds per pen was recorded daily. It comprised birds found dead and culled birds (due to very small size, leg deformities, lameness or abnormalities). The cumulative mortality was compared between strains on D31 for Ross 308, D46 for JA 757, and D39 for the other strains.

#### Body Condition

Body condition was assessed on D31 for Ross 308, D45 for JA 757, and D38 for the other strains. It consisted in scoring pododermatitis, hock burns, gait, cleanliness and plumage growth on 160 birds per strain × density conditions (40 randomly selected per pen, sex balanced). Scoring ranged from 0 to 2 points for each criterion, the lowest score indicating the best physical condition (Table 2). For the pododermatitis and hock burn scores, the broiler was picked up and its legs were shown to the trained observer, who then recorded the status of the worst of the 2 legs. At the same time, the observer scored breast cleanliness and plumage growth over the whole body except the breast area. The gait score was recorded by a second trained observer who watched the chicken from behind and gently encouraged it to walk (prompting it with a stick if necessary).

**Table 2:** Scoring of leg health, gait, breast dirtiness and plumage growth.

	Score 0	Score 1	Score 2
**Pododermatitis**	None or slight, very superficial lesions, slight discoloration on a limited area, slight hyperkeratosis or scarred skin	Moderate and severe discoloration, superficial lesions, darkened papillae	Severe dermatitis ulcers or scabs of significant size, signs of bleeding or severely swollen foot pads
**Hock burns**			
**Gait**	Normal gait, agile, with or without imbalance	Broiler walks more than 1.50 m but has difficulty in walking; limps	Broiler walks less than 1.50 m, and/or is severely lame, preventing movement
**Breast dirtiness**^1^	Clean—no stains on the feathers—nothing stuck to the skin or feathers	A few spots on the feathers, not covering the whole breast, no litter or stuck droppings	Most feathers are stained, and there may be dirty litter or stuck droppings
**Plumage growth**²	Fully covered (body, back, wings) or slightly uneven in one area	Two bare patches under the wings, but they are not joined	Very uneven plumage—or almost bare in one area, patches under the wings are joined

1 Scored if area is covered with feathers.

2 Except breast.

#### Behavior

Bird behavior was observed in 2 pens per strain × density conditions through video recordings by cameras placed above them (same pens as for hanging scales). Six short one min-videos (2 in the morning when the light was switched on, 2 in the middle of the day, and 2 in the evening before the light was switched off) were recorded on D30 for Ross 308, D44 for JA 757 and D37 for other strains. For each video, one scan (the first image) was used to record interactions with enrichments were observed by noting the number of broilers “Touching,” “Perching” or “Pecking” (Table 3). In order to precise the behavior registered (for instance, “Pecking,” video was played during few seconds. Percentages were calculated by dividing these numbers by the total number of broilers in the entire pen. In addition, for each video, a 4m² area free of enrichments, drinkers and feeders was observed (Figure 1, blue area, 2 m × 2 m). One scan (the first image) was used to record the number of broilers engaged in the following activities was recorded: “Standing,” “Foraging,” “Walking/Running,” “Grooming,” “Stretching,” “Flapping,” “Dustbathing” and “Being inactive” (Table 3). In order to precise these behaviors, video was played during few seconds. These behaviors were expressed as a percentage of the total number of birds in the observed area, which concerned around 40 individuals.

**Table 3:** Definition of the observed behaviors.

Behavior	Definition
**Touching**	Broiler touching a bale or pecking block, regardless of the bird's position (standing or lying down), or whether it is pecking or not
**Perching**	Bird perched on bale, scale or block, regardless of its activity
**Pecking**	Bird pecking a bale or a block (not its residues on the ground), regardless of its position in relation to it (perched or touching)
**Standing**	Bird standing
**Foraging**	Bird scratching or pecking the ground
**Walking or running**	Bird moving more than 3 steps
**Grooming**	Bird with its head in its feathers, cleaning them or scratching itself (excluding dust bath)
**Stretching**	Bird stretching a leg, wing or both
**Flapping**	Bird flapping its wings
**Dustbathing**	Bird lying down, stirring the litter and shaking
**Being inactive**	Bird standing or lying down, not doing any particular action (may move its head)

#### Litter quality

On D32 for Ross 308, D46 for JA 757, and D39 for other strains, litter quality was assessed using the Welfare Quality protocol (Welfare Quality, 2009) in each pen, with visual scoring of 3 areas per pen. Scores, described in Table 4, were expressed as the mean of the 3 areas per pen. In addition, 18 core samples were collected at different areas per pen and pooled together. A sample of 1 kg was analyzed for dry matter content.

**Table 4:** Scores for litter quality.

Score	Definition
**0**	Completely dry and flaky, i.e., easy to move with a foot
**1**	Dry but not easy to move with a foot
**2**	Leaves imprint of foot and will form a ball if compacted, but ball does not stay together well
**3**	Sticks to boots and readily stays in a ball if compacted
**4**	Sticks to boots once the (compacted) crust is broken

#### Consumption of bales and pecking blocks

Pecking blocks and bales (alfalfa and straw) were weighed in each pen when renewed and on D31 for Ross 308, D46 for JA 757, and D39 for the other strains. Consumption was estimated as the difference between the initial and final weight of blocks and bales. It included actual ingestion but also dispersion of particles into the litter due to the birds’ pecking and scratching behaviors.

### Statistical Analysis

The statistical unit was the pen, except for body condition characteristics and manual BW for which the statistical unit was the bird. Statistical analyses were conducted in R Studio (Team, 2020). The multilevel linear model (*geeglm*: generalized estimating equations) was used for variance analysis of the data, after having visually validated the distributions of the model residuals for normality. Strain, density, and strain by density interactions were included in the model as fixed effects. The pen was included as a random effect when appropriate. For behavioral data, the number of the video recording was added as a random effect. When interaction was significant, posthoc analyses were carried out by a Student-Newman-Keuls test with Benjamini-Hochberg adjustment for multiple comparisons. When a main effect or the interaction was not significant, it was removed from the model. For all analyses, differences were considered significant when *P* ≤ 0.05.

## RESULTS

Means by strain, density, and significance of effects are shown in Tables 5–9. Detailed data per strain × density conditions are in the supplementary file (Table 9). Automatic scale data indicated that the target weight (1.80–1.95 kg) was reached very close to the expected age (Table 1). Depending on BW and mortality during the rearing period, densities at these ages were 37.1–39.3 kg/m² for HD and 28.6–29.1 kg/m² for LD, close to the expected values (37 kg/m² for HD, and 29 kg/m² for LD pens).

**Table 5:** Mean values (± standard deviations) of body weight (BW, manual, g) on D32, total (M_1_) and late (M_2_) mortality rates, and significance of strain, density and strain × density effects.

	Strain^1^						Density^2^				Significance of effects		
	Ross 308	Redbro	Rustic Gold	Ranger Classic	JA 787	JA 757		HD	LD	Strain (S)		Density (D)	S×D
**BW32 (g)**^3^	2,206^a^ (19)	1,517^b^ (13)	1,465^b,c^ (8)	1,445^c^ (10)	1,441^c^ (5)	1,140^d^ (5)		1,546 (7)	1,542 (5)	<0.001		0.515	0.423
**M_1_ (%)**^4^^,^^5^	5.28^b^ (0.54)	5.85^b^ (0.22)	4.38^b^ (0.36)	3.71^b^ (0.19)	5.28^b^ (1.17)	10.41^a^ (0.46)		6.13 (0.42)	5.51 (0.23)	<0.001		0.242	<0.001
**M_2_ (%)**^4^^,^^6^	2.03^a^ (0.24)	1.40^a,b^ (0.15)	1.36^a,b^ (0.16)	1.03^b^ (0.11)	1.12^a,b^ (0.35)	1.33^a,b^ (0.22)		1.42 (0.13)	1.34 (0.12)	0.017		0.697	0.511

1 Having different superscripts within a row indicates a significant difference between the groups (*P* < 0.05).

2 HD: high density, LD: low density.

3 N = 104 birds per strain × density level.

4 N = 4 pens per strain × density level.

5 From D0 to D32 for Ross 308, from D0 to D46 for JA 757 and from D0 to D39 for other strains, as a percentage of animals on D0.

6 From D11 to D32 for Ross 308, from D11 to D46 for JA 757 and from D11 to D39 for other strains, as a percentage of birds on D11.

### Body Weight

As expected, there was a significant effect of the strain on BW on D32. Ross 308 broilers were 45%–53% heavier than Redbro, Rustic Gold, Ranger Classic, and JA 787 broilers, and 93% heavier than JA 757 broilers (Table 5). Stocking density did not influence BW on D32.

### Mortality

A significant interaction between strain and density was found on mortality rate at target weight (total mortality rate, Table 5). This was due to a high mortality in JA 757 broilers, especially at HD (12.5%, significantly higher than all other strain × density conditions). Mortality was also high for LD-JA 757 (8.3%) but was only significantly higher when compared with LD-Ranger Classic (3.4%). However, this high mortality rate in JA 757 mainly occurred at the beginning of the rearing period (before D10) when some chicks had to be culled, probably due to the heterogeneous chick quality observed at 1 d old in this batch. Indeed, the mortality from D11 to slaughter (late mortality) was not significantly different between JA 757 and the other strains. The only significant difference observed on late mortality between strains was due to a higher mortality rate in Ross 308 than Ranger Classic. Finally, stocking density did not affect either total or late mortality rates.

### Body Condition

Table 6 shows the percentage of broilers that were scored 0 for each body condition indicator measured. At 31 d of age, Ross 308 birds were not feathered on the breast, so they could not be scored for cleanliness. A significant interaction between strain and density was found for pododermatitis due to a significant strain effect only in HD pens. In HD pens, the proportion of chickens without pododermatitis was lower in Ross 308 (65.0%), Redbro (72.2%), and JA 787 (67.5%) than in the other strains (89.6%, 88.3%, and 89.4% for Rustic Gold, Ranger Classic, and JA 757, respectively). However, scores for the 6 strains were comparable in LD pens. Indeed, the reduction in stocking density greatly improved pododermatitis scores in Ross 308, Redbro, and JA 787 (rising from 68.2% of broilers free of pododermatitis in HD pens to 93.9% in LD pens, taking an average of the 3 strains). The improvement was only moderate in the other strains (from 89.1% in HD pens to 97.6% in LD pens). Regardless of the strain, a lower density was also associated with a better body condition, with fewer hock burn lesions and better scores for gait and breast cleanliness (Table 6).

**Table 6:** Mean percentage (± standard deviation) of broiler chickens that were scored 0 (no disorders) for pododermatitis, hock burns, gait, breast dirtiness and plumage growth (D31 for Ross 308, D45 for JA 757 and D38 for other strains), and significance of strain, density and strain × density effects.

	Strain^1^						Density^2^		Significance of effects		
Criterion	Ross 308	Redbro	Rustic Gold	Ranger Classic	JA 787	JA 757	HD	LD	Strain (S)	Density (D)	S×D
**Pododermatitis**	78.8^b^ (1.8)	84.5^b^ (2.9)	93.9^a^ (1.9)	92.7^a^ (1.2)	81.1^b^ (2.8)	93.5^a^ (2.1)	78.6 (1.7)	96.2 (0.6)	0.020	<0.001	<0.001
**Hock burn**	66.9^a^ (3.8)	63.9^a^ (5.5)	68.6^a^ (2.7)	78.7^a^ (3.1)	79.6^a^ (3.3)	76.6^a^ (4.2)	62.9 (2.8)	81.8 (1.4)	0.107	<0.001	0.415
**Gait**	54.4^c^ (3.7)	90.9^a,b^ (2.0)	85.7^b^ (2.1)	99.1^a^ (0.4)	97.3^a^ (0.7)	98.8^a^ (0.7)	85.2 (1.1)	90.2 (1.2)	<0.001	0.003	0.200
**Breast dirtiness**^3^	-	6.1^b^ (1.3)	9.1^b^ (1.6)	25.2^a^ (2.9)	20.3^a^ (3.2)	24.8^a^ (2.2)	10.7 (1.3)	23.2 (1.6)	<0.001	<0.001	0.089
**Plumage growth**	0.0^d^ (0.0)	22.6^c^ (2.5)	27.4^b^ (2.9)	29.6^b^ (2.6)	31.1^b^ (2.8)	67.9^a^ (3.0)	27.7 (1.4)	31.8 (1.6)	<0.001	0.060	0.178

1 Having different superscripts within a row indicates a significant difference between the groups (*P* < 0.05).

2 HD: high density, LD: low density.

3 Scored only for broilers with a feathered breast. N=160 birds per strain × density level.

JA757, JA787, and Ranger Classic had the highest percentages of score 0 (i.e., the best score) for gait and breast cleanliness. Ross 308 had the lowest one for gait, and Redbro and Rustic Gold were intermediate for gait and breast cleanliness. Plumage quality varied greatly between strains, with the highest percentages of score 0 for JA757, the lowest for Ross 308 and intermediate values for the other 4 strains. Ranger Classic broilers had similar results to Rustic Gold, but with a better gait score and cleaner breasts. Conversely, JA 787 broiler chickens had more pododermatitis than JA 757 and Ranger Classic chickens.

### Behavior

Perching behavior was observed in all the videos, whatever the density or the strain. There were up to 7 broilers perched on the scale simultaneously, a maximum of 4 birds on alfalfa or straw bales, but usually from one to 3 (except for Ross 308 birds, that never perched on them), and up to 2 at a time on the pecking block. These figures represent a maximum of 5% of broilers perched at the same time in each pen during the observation period.

#### Interactions with enrichments

An interaction between strain and density was found for the percentage of broilers interacting with enrichments (Table 7), especially perching and pecking, due to minor differences between Redbro and JA 757 birds at HD but not LD. At HD, JA 757 birds interacted more with enrichments than Redbro birds (respectively, 8.85% vs. 7.01% of broilers observed interacting with enrichments, *P* = 0.021), whereas there was no significant difference between the 2 strains at LD (8.15% for JA 757 broilers vs. 8.41% for Redbro broilers, *P* = 0.721). Beyond this interaction, a strain effect was observed on the percentage of broilers interacting with, perching on or pecking at enrichments (bales, pecking block and scale) whatever the density. Fewer interactions with enrichments (especially perching activity) were observed in Ross 308 than in all other strains, and JA 757 birds were no different from the other tested strains. The Rustic Gold birds, however, were closer to the Ross 308 broilers, none of which were observed perched on bales (whether at 30 or 60 cm high); they only perched on the pecking block and scale. Pecking enrichment interactions were 5 times more frequent in JA 757 than in Ross 308, the other strains being in between (Table 7). Stocking density did not significantly affect any of the observed behaviors.

**Table 7:** Mean broiler activity (± standard deviation; as a percentage of birds in the area) and interaction with enrichments (as a percentage of birds in the pen) at target weight (Ross 308 on D30, JA 757 on D44 and all others on D37), and significance of strain, density and strain × density effects.

	Strain^1^						Density^2^		Significance of effects		
	Ross 308	Redbro	Rustic Gold	Ranger Classic	JA 787	JA 757	HD	LD	Strain (S)	Density (D)	S×D
**Interacting with enrichments**^3^	5.27^c^ (0.07)	7.71^a,b^ (0.31)	6.72^b^ (0.25)	7.50^a,b^ (0.15)	7.45^a,b^ (0.15)	8.50^a^ (0.03)	6.74 (0.10)	7.31 (0.11)	<0.001	0.069	<0.001
**Touching**	3.48^a^ (0.08)	3.86^a^ (0.20)	3.65^a^ (0.26)	3.64^a^ (0.12)	3.56^a^ (0.14)	3.88^a^ (0.13)	3.45 (0.05)	3.72 (0.13)	0.668	0.059	0.538
**Perching**	1.55^c^ (0.08)	3.13^a,b^ (0.12)	2.71^b^ (0.11)	3.15^a,b^ (0.12)	2.98^a,b^ (0.05)	3.33^a^ (0.10)	2.71 (0.05)	2.77 0.07)	<0.001	0.785	<0.001
**Pecking**	0.25^d^ (0.06)	0.73^b,c^ (0.09)	0.36^c,d^ (0.09)	0.72^b,c^ (0.03)	0.91^b^ (0.04)	1.29^a^ (0.25)	0.58 (0.04)	0.70 (0.09)	<0.001	0.393	0.014
**Standing**	10.06^c^ (0.70)	24.43^a,b^ (1.76)	18.07^b,c^ (1.64)	23.86^a,b^ (1.45)	31.00^a^ (1.15)	23.13^a,b^ (1.21)	21.07 (0.88)	22.10 (0.68)	<0.001	0.490	<0.001
**Foraging**	5.19^b^ (0.44)	10.06^b^ (1.24)	7.82^b^ (1.12)	9.07^b^ (0.56)	9.37^b^ (0.43)	15.81^a^ (1.19)	8.78 (0.46)	8.77 (0.58)	<0.001	0.980	0.810
**Walking/running**	1.84^a^ (0.32)	2.32^a^ (0.27)	2.49^a^ (0.45)	3.22^a^ (0.37)	4.20^a^ (0.30)	1.77^a^ (0.54)	2.76 (0.27)	2.73 (0.16)	0.060	0.876	0.333
**Grooming**	8.42^a^ (1.46)	8.66^a^ (0.42)	8.22^a^ (0.38)	11.33^a^ (1.21)	9.99^a^ (0.21)	13.72^a^ (2.75)	9.50 (0.63)	8.72 (0.94)	0.111	0.559	0.136
**Being inactive**	78.97^a^ (0.98)	71.98^b,c^ (1.36)	73.99^b,c^ (2.15)	67.10^c,d^ (0.82)	64.41^d,e^ (1.51)	59.15^e^ (1.56)	71.47 (0.98)	72.03 (0.65)	<0.001	0.745	0.229

1 Having different superscripts within a row indicates a significant difference between the groups (*P* < 0.05);

2 HD: high density, LD: low density.

3 Interacting with enrichments means total of Perching on bales and the scale, Touching the bales and the block, Pecking at bales and the block. N = 2 pens x 6 short videos per strain × density level.

#### General Activity

A strain by density interaction was found for standing, as there was a higher percentage of JA 787 broilers standing at LD than at HD (36.8% vs. 25.2%), but there was no density effect for the other strains. The percentage of standing broilers was 2.3–3.1 times lower in Ross 308 than in Redbro, Ranger Classic, JA 787, and JA 757 strains, whatever the density. The percentages of broilers observed foraging or being inactive also differed significantly between strains. Foraging behavior was 3 times more frequent in JA 757 than Ross 308, other strains showing similar intermediate frequency values between 1.5 and 1.9 more than Ross 308. The percentage of inactive broilers globally followed a gradient in relation to the birds’ growth rate (∼+0.5% of inactive birds per gram of ADG). There were 20% more inactive Ross 308 broilers than JA 757 broilers, and it is noteworthy that Ranger Classic and JA 787 showed intermediate values quite close to those of JA 757.

The percentages of broilers observed grooming (9.1%) and walking/running (2.7%) were not affected by either strain or density. Finally, some behaviors important for animal welfare, such as dustbathing (0.2%), flapping (0.1%), and stretching (0.9%), were observed in very few broilers, so neither density nor genetic effects could be analyzed.

### Litter

Both strain and density affected litter quality (Table 8). In LD pens, litter was significantly drier and of a better quality than in HD pens. The percentage of dry matter in litter decreased with growth rate (−0.2% of dry matter per gram of ADG), but the difference was significant only between the JA 757 strain, which had the highest value, and Ross 308 with the lowest values, all other strains having intermediate values. The quality score was better for Ranger Classic and JA 787 and worse for Ross 308, Redbro, Rustic Gold, and JA757.

**Table 8:** Mean (± standard deviation) litter dry matter content and quality score, consumption of bales (straw and alfalfa) and blocks, and significance of strain, density and strain × density interaction effects on D31 for Ross 308, D46 for JA 757 and D39 for other strains.

	Strain^1^						Density^2^		Significance of effects		
	Ross 308	Redbro	Rustic Gold	Ranger Classic	JA 787	JA 757	HD	LD	Strain (S)	Density (D)	S × D
**Litter dry matter (%)**	47.8^b^ (0.9)	50.1^a,b^ (0.6)	51.3^a,b^ (0.7)	51.3^a,b^ (0.9)	52.0^a,b^ (1.3)	54.2^a^ (1.8)	48.6 (0.7)	53.3 (0.6)	0.027	<0.001	0.404
**Litter quality score**^3^	2.5^a^ (0.1)	2.7^a^ (0.1)	2.4^a^ (0.1)	1.6^b,c^ (0.1)	1.5^c^ (0.1)	2.1^a,b^ (0.1)	2.5 (0.1)	1.7 (0.1)	<0.001	<0.001	0.190
**Block consumption (g/bird)**	2.1^c^ (0.2)	6.0^a^ (0.4)	2.1^c^ (0.2)	4.5^b^ (0.1)	6.5^a^ (0.5)	7.4^a^ (0.4)	5.0 (0.2)	4.5 (0.2)	<0.001	0.052	0.516
**Bale consumption (g/bird)**	155^c^ (11)	451^b^ (19)	508^b^ (26)	433^b^ (8)	535^b^ (35)	974^a^ (63)	447 (13)	577 (24)	<0.001	<0.001	<0.001

1 Having different superscripts within a row indicates a significant difference between the groups (*P* < 0.05).

2 HD: high density, LD: low density.

3 Mean of the 3 areas per pen, from 0 to 4, 0 being a completely dry and flaky litter. N=4 pens per strain × density level.

### Consumption of Bales and Pecking Blocks

Among all the enrichments, only alfalfa bales placed on the upper level in JA 757 pens were renewed. The mean consumption per pen was 1.2 kg (range 0.1; 2.6) out of the initial 8 kg for blocks, 4.2 kg (range 1.5; 8.9) out of the initial 15 kg for straw bales and 2.3 kg (range 0.3; 5.9) out of the initial 20 kg for alfalfa bales on the lower level. Considering all strains except JA 757, 4.8 kg (range 0.2; 12.3) out of the initial 20 kg for alfalfa bales on the upper level was consumed per pen, whereas 14.6 kg (range 9.3, 23.6) was consumed per JA 757 pen over the rearing period.

An interaction between strain and density was found for bale consumption due to a significant density effect only in JA 757 pens: consumption was higher in LD (1198 g/bird) than in HD pens (750 g/bird). There was no density effect on bale consumption in the other strains (Table 8).

Block consumption was higher for JA757, JA787, and Redbro than Ranger Classic (which was intermediate) and Ross 308 and Rustic Gold (which was low). JA757 also stood out by the highest consumption of bales, Ross 308 by the lowest, while the other 4 strains were intermediate.

## DISCUSSION

The aim of this study was to identify how growth rate and stocking density influence broiler behaviors, health, litter quality and consumption of enrichments in order to propose a combination of strain and density that improves bird welfare.

Comparing strains with different growth rates implies confusion between age and weight effects (Santos et al., 2022). Here it was decided to compare strains with different growth rates at the same target weight, and thus at different ages, to have a global view of the production cycle, which differs between strains. For some indicators such as bale and block consumption or litter quality, the differences observed may therefore be due to behavioral differences between strains as well as differences in rearing periods.

Some indicators were affected by density whatever the strain (hock burn prevalence, gait, breast cleanliness, litter quality). Conversely, body weight, mortality, gait, breast cleanliness, plumage growth, proportion of birds foraging or being inactive, litter quality and block consumption were affected by strain whatever the density. Interactions between growth rate and density were found as well. This is discussed below in specific sections.

A point of attention is that we cannot fully exclude that strain effects were also confounded by room effects, as one single strain was represented per room, for technical reasons (strains requiring different regimes given their different growth rates). However, previous studies in the same building showed that differences between rooms were almost null, then we have every reason to believe that this was still the case here.

### Interactions Between Strain and Density

The most important interaction between strain and density was found for pododermatitis. A low density positively affected strains with the highest prevalence of pododermatitis. Strain differences observed at high density disappeared at low density. High stocking densities are considered a risk for contact dermatitis, which has been related to the adverse effect on litter quality (EFSA AHAW Panel et al., 2023). Except for this interaction, limited interactions were found for other variables (perching, standing behavior and bale consumption), indicating that the differences between strains were globally observed whatever the density, and conversely.

### Differences Among Strains

As expected, the target weight (1.80–1.95 kg) was reached the earliest by Ross 308 broilers (29 d) and the latest by JA 757 (44 d), other strains showing intermediate values (between 37 and 41 d).

The high mortality in JA 757 chicks observed during the first days of rearing did not have any clear explanation except the relatively poorer chick quality of this particular flock. All the strains were reared in the same conditions (environment, nutrition, lighting program), but it was not possible to control parent stock source or age, hatching conditions, and chick transport conditions, which can all affect chick quality (Bergoug et al., 2015) and thus early mortality. Apart from this early period, mortality was low for all strain × density combinations.

At the same BW, the mean litter quality was poorer for Ross 308 (despite the addition of litter material during rearing and despite the shorter rearing period), Redbro, and Rustic Gold, and better for Ranger Classic and JA 787. The litter quality score for JA 757 was worse than for JA 787, probably due to the longer rearing period for JA 757, decreasing visible litter quality. Litter humidity increased with increased growth rate, which probably partly explains the better leg conditions in the slower-growing strains, as contact dermatitis is caused by prolonged contact of the skin with humid and soiled litter (Bessei, 2006). With the fastest growth rate, Ross 308 birds had the highest percentages of broilers with pododermatitis and gait problems. Almost half of the Ross 308 broilers had a gait disorder. A previous study showed that, even reared under organic conditions, the prevalence of lameness (score > 0/5) was even higher for Ross 308 (90%, with 20% prevalence of severe lameness) (Eriksson et al., 2010). Our score 0 for gait included birds without imbalance or with moderate imbalance, but most Ross 308 broilers that scored 0 had an imbalanced gait, whereas in other strains, the 0 score was due to an absence of imbalance (personal observation). This can explain the higher prevalence in Eriksson et al.’s study than in ours (Eriksson et al., 2010).

Interestingly, the prevalence was higher for hock burns than for pododermatitis, whatever the strain × density conditions. This was also reported by Tahamtani et al. (2018), who examined the prevalence in 60 Danish broiler flocks. Pododermatitis depends mostly on litter quality (related to multiple factors), and hock dermatitis is linked to activity levels (Haslam et al., 2006; Dawson et al., 2021). Indeed, increased activity, in addition to reduced contact time of legs and hocks with litter, increases litter ventilation by the birds’ scratching and foraging activities. This may in turn reduce the prevalence of pododermatitis in broilers and improve plumage cleanliness due to a drier litter (Riber et al., 2018). This is consistent with the fact that JA 757, JA 787, and Ranger Classic strains, which displayed very few abnormal gaits, were also the cleanest (in concordance with the study by Ghayas et al. (2021) in which slower-growing broilers had a better feather cleanliness score than fast-growing broilers) and the most active. Moreover, Rustic Gold, whose behaviors were globally close to Ross 308, had quite a high prevalence of hock burns (more than 30%). Indeed, at the same BW, these 3 strains (JA 757, JA 787, and Ranger Classic) had a lower percentage of inactive birds (59%–67%) than the Ross 308 and Rustic Gold (74%–79%) strains, more standing birds (23%–31% vs. 10%–18%) and more foraging birds (9%–16% vs. 5%–8%). Globally, activity followed an inverse gradient to the broilers’ growth rate: the slower they grew, the more active they were at the target weight. Other studies have already shown that slower-growing broilers walked, stood, and foraged more, and ate and sat less than fast-growing broilers (Dixon, 2020; Rayner et al., 2020; Dawson et al., 2021; van der Eijk et al., 2022). Slower-growing broilers also displayed more play behaviors, such as frolicking and sparring (Baxter et al., 2021). This was not observed in our study, but should be further investigated, especially with enrichments in the environment.

To summarize, as growth rate increased, we observed more birds that were inactive, and decreased foraging. This was combined with an increase in litter humidity, deterioration in breast cleanliness, and an increased sensitivity to pododermatitis. Nevertheless, other factors appear to be involved for this last indicator as JA787 was just as affected as Ross 308 and Rustic Gold, especially at the high density.

Another behavioral difference between strains was that Ross 308 perched only on the pecking block or scale, but not at all on bales, whether at 30 cm or 60 cm. This explains why in this strain, perching did not exceed 1.6% whereas it was twice that for Redbro, Ranger Classic, JA 787, and JA 757 (3.0%–3.3%). These findings are consistent with van der Eijk et al. (2022), who observed that slower-growing broilers made better use of enrichments by sitting on the bales, whereas fast-growing broilers did not. In previous studies, Ross 308 broilers were observed perching on a 19-cm-high straw bale, and on a platform 30 cm high when ramps were accessible next to the platform (Mocz et al., 2022; Mocz et al., 2023). This being so, providing perching facilities higher than 30 cm to Ross 308 broilers appears meaningless if no ramp is offered. It is thus important to propose enrichments suited to fast-growing strains, particularly for perching. Better use of enrichments by slower-growing strains may be explained by a higher activity level or by a different body conformation (De Jong and Gunnink, 2019) resulting in fewer problems for maintaining their balance or a better ability to fly or climb onto an enrichment. The higher frequency of bale and block pecking behavior in JA 757 and, to a lesser extent, Redbro, Ranger Classic, and JA 787 broilers is consistent with bale consumption observed in these strains. It could be attributed to a difference in foraging behavior between the strains, as the access to these enrichments was equivalent for all birds.

In our study, decreased growth rate was associated with better plumage growth. It should be noted that our plumage scores did not reveal plumage damage, but rather age-dependent plumage growth. On D31, Ross 308 birds were not fully covered in plumage; this was also the case, but to a lesser extent, on D45 for intermediate strains. Early plumage growth in broilers can provide effective protection against chills and better insulation against heat loss (Yalcin et al., 1997). Poor plumage growth can be source of discomfort, especially for the late stages (broilers chicken collection and transport for slaughterhouse), as feathers help to form a tough outer skin that provides an insulating layer for birds to trap warm air and protect birds from external mechanical damage (Lingham, 2019).

### Stocking Density

The HD condition in our study was similar to the situation encountered in many European farms where stocking density varies between 33 and 42 kg/m², with an average value of 36.3 kg/m² (European Commission, 2017) for a final BW of 2 kg. The LD condition met the expectations of 30 kg/m² for a final BW of 2 kg specified in the European Chicken Commitment (2019).

No significant differences in BW (whether at the target weight or any age, data not shown) or cumulative mortality rate were found between the 2 stocking densities. This is consistent with the literature for mortality (Ventura et al., 2010; Sekeroglu et al., 2011; Mocz et al., 2022). Results in the literature on BW are more contrasted. No effect of stocking density on BW was found by Buijs et al. (2009) on D39 with densities from 6 to 56 kg/m² nor by Ventura et al. (2010) on D43 with densities from 21 to 47 kg/m². Conversely, Mocz et al. (2022) and Sekeroglu et al. (2011) found a negative effect of high density on body weight. However, the former compared their birds on D25 (densities of 31 and 41 kg/m²). Moreover, at a comparable age and weight, the high-density condition used by Sekeroglu et al. (2011) (31 kg/m²) was close to our LD condition. In addition, in our study, the same diet was given to all strains, formulated to fulfil Ross 308 requirements and thus exceeding those of the other strains, which may have compensated for any effect of high density on BW in our study.

The reduction in stocking density greatly improved leg conditions in terms of pododermatitis (only for the strains having more problems: Ross 308, Redbro, and JA 787) and hock burn lesions. Probably as a consequence, LD broilers had better gait scores than HD ones. These effects are likely to be related to the better litter quality in LD than HD pens (humidity and score), which also contributed to the better cleanliness of LD broilers. Indeed, a high stocking density can lead to wetter litter with a higher ammonia content, increasing the prevalence of contact dermatitis (Dozier et al., 2005; Buijs et al., 2009; Mocz et al., 2022). A better walking ability, lower prevalence of contact dermatitis and better litter quality with lower stocking densities are in concordance with a recent study (van der Eijk et al., 2022).

Our observations did not highlight any difference between LD and HD birds in activity or interactions with enrichments. The lack of behavioral difference is consistent with the fact that the consumption of bales and blocks was similar for both LD and HD pens except for JA 757, where consumption of bales was higher in the LD than the HD pen. It is possible that the difference in density was not large enough to induce visible effects in the relatively small experimental pens we used, in agreement with van der Eijk et al. (2022), who found that stocking density did not affect enrichment use in either semi-commercial conditions such as ours or experimental facilities.

Previous studies have reported that stocking density affects broiler behavior, but findings are not always consistent. A study across 114 commercial flocks did not find a major influence of density on chicken welfare (Dawkins et al., 2004). In other studies for instance, locomotion was found to decrease between stocking densities of 34 and 40 kg/m² in Hall (2001) and between 24 and 42 kg/m² in van der Eijk et al. (2022). Conversely, no effect of stocking density on activity was found by Collins (2008) in an experiment comparing a stocking density of 26 kg/m² with that of 43 kg/m², nor by McLean et al. (2002) comparing 28, 34 and 40 kg/m² stocking densities. The same discrepancy was found on the effect of density on litter-directed behavior, with a negative effect of density in Buijs et al. (2009), Hall (2001), and van der Eijk et al. (2022), but no effect in other reported studies (McLean et al., 2002; Febrer et al., 2006). It should be noted that although statistically significant, the differences among stocking densities in these studies were quite small. For instance, in van der Eijk et al. (2022), the locomotor and comfort/foraging behaviors were, respectively, 0.5% and 1.5% more frequent in LD pens than in HD pens. Another explanation for the lack of difference in terms of behavior in our study may be that, despite a 22% reduction in density between LD and HD, the density still remained much higher than the 11 kg/m² recently recommended by EFSA (EFSA AHAW Panel et al., 2023) to give broilers the opportunity to fully express their behaviors.

Moreover, even if no difference in either activity or interaction with enrichments was highlighted between LD and HD birds, it is possible that high density affected disturbance during resting, but that was not observed and should be further investigated.

Finally, observation by short videos was probably not best suited to furtive or rare behaviors such as dustbathing, flapping and stretching, which should also be further investigated. Data collection by continuous sampling or more frequent scan sampling could address issues of this kind. AI programs development could also help to detect automatically some behaviors and thus improve the speed of analysis of videos, which is the most important limit for this strategy right now.

### Recommendations About Enrichments Tested in the Experiment

Whatever the strain × density conditions, no pecking block had to be replaced during the rearing period. For the whole rearing period, block consumption represented an average of 8 g per bird in the pens where consumption was the highest (JA 757). When considering only the satisfaction of foraging behavior, a total quantity of 8 kg of pecking block at most for 1,000 birds could be recommended for this strain. For the other strains, the recommendation could be lower, from 2 kg for 1,000 Ross 308 broilers to 7 kg for 1,000 JA 787 broilers. However, there should be more than one block for 1,000 birds, considering that the blocks must be accessible to the majority of birds at the same time. With the setting presented (2 alfalfa bales and one straw bale) in our study, bale consumption throughout the rearing period represented an average of 25 kg of straw plus alfalfa per pen in the JA 757 pens, which had the highest consumption, 4 kg in Ross 308 pens and around 12 kg for the other strains. According to these results, for every 1,000 birds, 98 kg of bales (i.e., 6 bales), 16 kg (i.e., 1 bale) and 47 kg (i.e., 3 bales) could be provided for JA 757, Ross 308, and the other broiler strains, respectively. However, recommendations in terms of bale quantity have to be adjusted depending on the compression and size of bales (smaller bales will disintegrate faster due to their size and accessibility). Moreover, if no other perching material can be offered to Ross 308 broilers, bale height should be lower (<18 cm) than the bales in this experiment, which were not used for perching. It can be considered that a broiler needs a surface area of 320–500 cm² to perch on (Norring et al., 2016; Spindler et al., 2016; Kaukonen et al., 2017). In our study, the bales, pecking block, and scale represented a total surface area of 6,187 cm² for perching (3,187 cm² for Ross 308 considering that they did not perch on the bales). This means that a maximum of 12–19 broilers were able to perch at the same time (6–10 in Ross 308 pens), i.e., 4%–8% of broilers in the pens (2%–4% for Ross 308) at the target BW. We observed a range of broiler perching percentages from 1.55% (Ross 308) to 3.33% (JA 757). This means that almost all the area provided for perching was used by broilers, except for Ross 308 due to the lack of ramps. If we aim to provide enough perching space on commercial poultry farms to enable a minimum of 3% of broilers to perch simultaneously, for example, an additional 0.96–1.50 m² of elevated surface (bales, blocks, scales or platforms) would be required for 1,000 birds. This is in accordance with the French animal welfare labelling scheme “Etiquette Bien-Être Animal,” which requests 1 m² of perching surface for every 1,000 broilers. Twice this area is required for higher welfare standards (Association-Etiquette-Bien-Être-Animal, 2021). Additional elevated surfaces would probably increase the percentage of broilers perching simultaneously, thus improving their welfare. This should be further investigated to clarify the minimal surface area of elevated structures to provide in order to fulfil this behavioral need. Again, it is important to provide ramp access—especially for Ross 308 birds. For instance, a maximal angle of 25° is recommended by EFSA (EFSA AHAW Panel et al., 2023).

## CONCLUSION

In conclusion, the results from the present study highlight the importance of lowering the growth rate to improve broiler welfare in conventional rearing conditions. Whatever the decrease in growth rate (compared with Ross 308), it significantly improved many welfare indicators such as gait score, plumage growth, standing, inactivity, perching and pecking enrichments. Other indicators related to better welfare need a greater decrease in growth rate to be significantly improved (e.g., pododermatitis prevalence in HD, foraging behavior, litter quality). Lowering stocking density (37 kg/m² vs. 29 kg/m² at the target weight) did not influence BW, mortality or behaviors, but improved litter quality and reduced breast cleanliness, hock burn prevalence and gait. Sufficient enrichments need to be provided and must be tailored to broiler morphology to meet behavioral needs. Other management measures to control litter quality must also be taken into consideration to improve broiler welfare.

## DISCLOSURES

The authors declare that they have no known competing financial interests or personal relationships that could have appeared to influence the work reported in this paper.

## Appendix Supplementary materials

Image, application 1

Image, application 2

## Footnotes

Supplementary material associated with this article can be found in the online version at doi:10.1016/j.psj.2024.103993.
