# Supplementary material for: Effects of strain and stocking density on leg health, activity, and use of enrichments in conventional broiler chicken production
Source: Poult Sci. 2024 Jun 27;103(9):103993. doi: 10.1016/j.psj.2024.103993 (PMC11298928; doi:10.1016/j.psj.2024.103993)
Supplement: Supplementary file 1 [file mmc1.docx]

Table 9: Supplementary file: data per strain x density conditions: mean body weight (BW, manual, g, at D32), total (M_1_) and late (M_2_) mortality rates, percentage of broiler chickens that were scored 0 (no disorders) for pododermatitis, hock burns, gait, breast dirtiness and plumage growth (D31 for Ross 308, D45 for JA 757 and D38 for other strains), broiler activity (as a percentage of birds in the area) and interaction with enrichments (as a percentage of birds in the pen) at target weight (Ross 308 on D30, JA 757 on D44; others on D37), litter dry matter content and quality score, consumption of bales (straw and alfalfa) and blocks on D31 for Ross 308, D46 for JA 757 and D39 for other strains. HD: high density, LD: low density.

|  | **Ross 308** | | **Redbro** | | **Rustic Gold** | | **Ranger Classic** | | **JA 787** | | **JA 757** | |
| --- | --- | --- | --- | --- | --- | --- | --- | --- | --- | --- | --- | --- |
|  | **HD** | **LD** | **HD** | **LD** | **HD** | **LD** | **HD** | **LD** | **HD** | **LD** | **HD** | **LD** |
| **BW32** | 2186 | 2225 | 1503 | 1532 | 1460 | 1470 | 1450 | 1439 | 1432 | 1450 | 1143 | 1138 |
| **M1**^1^ | 5,1 | 5,4 | 5,5 | 6,2 | 4,8 | 4,0 | 4,0 | 3,4 | 4,9 | 5,7 | 12,5 | 8,3 |
| **M2**^2^ | 1,91 | 2,14 | 1,46 | 1,34 | 1,48 | 1,23 | 0,95 | 1,11 | 1,57 | 0,67 | 1,03 | 1,56 |
| **Pododermatitis** | 65,0 | 92,5 | 72,2 | 97,0 | 89,6 | 98,2 | 88,3 | 97,0 | 67,5 | 95,1 | 89,4 | 97,5 |
| **Hock burn** | 51,3 | 82,5 | 58,5 | 68,9 | 58,5 | 78,7 | 72,8 | 84,3 | 68,7 | 90,7 | 67,7 | 85,6 |
| **Gait** | 49,4 | 59,4 | 87,2 | 94,5 | 82,3 | 89,0 | 98,8 | 99,4 | 95,8 | 98,8 | 97,5 | 100,0 |
| **Breast dirtiness**^3^ | - | - | 3,7 | 8,5 | 4,3 | 14,0 | 14,8 | 35,5 | 12,7 | 27,8 | 18,0 | 30,0 |
| **Plumage growth** | 0 | 0 | 20,1 | 25,0 | 20,1 | 34,8 | 28,4 | 30,7 | 30,1 | 32,1 | 67,7 | 68,1 |
| **Interacting with enrichments**^4^ | 5,24 | 5,30 | 7,01 | 8,41 | 6,41 | 7,03 | 6,94 | 8,07 | 7,41 | 7,49 | 8,85 | 8,15 |
| **Touching** | 3,28 | 3,67 | 3,62 | 4,09 | 3,58 | 3,72 | 3,41 | 3,87 | 3,34 | 3,78 | 3,52 | 4,24 |
| **Perching** | 1,73 | 1,36 | 2,84 | 3,42 | 2,59 | 2,82 | 2,91 | 3,38 | 3,11 | 2,85 | 3,87 | 2,79 |
| **Pecking** | 0,24 | 0,27 | 0,55 | 0,90 | 0,23 | 0,49 | 0,61 | 0,83 | 0,97 | 0,86 | 1,46 | 1,11 |
| **Standing** | 10,92 | 9,21 | 25,53 | 23,32 | 18,66 | 17,49 | 23,86 | 23,86 | 25,22 | 36,78 | 24,59 | 21,66 |
| **Foraging** | 5,39 | 4,99 | 10,89 | 9,23 | 6,97 | 8,68 | 8,60 | 9,54 | 9,48 | 9,25 | 16,44 | 15,18 |
| **Walking / running** | 2,27 | 1,41 | 1,89 | 2,76 | 2,86 | 2,11 | 3,19 | 3,24 | 4,03 | 4,35 | 1,43 | 2,11 |
| **Grooming** | 6,43 | 10,41 | 10,59 | 6,73 | 9,08 | 7,35 | 11,55 | 11,11 | 9,79 | 10,19 | 17,13 | 10,31 |
| **Being inactive** | 79,95 | 78,00 | 69,26 | 74,69 | 74,04 | 73,94 | 67,26 | 66,93 | 65,91 | 62,90 | 55,28 | 63,02 |
| **Litter dry matter (%)** | 46,7 | 48,9 | 48,7 | 51,5 | 48,3 | 54,3 | 48,7 | 54,0 | 48,9 | 55,0 | 51,2 | 56,5 |
| **Litter quality score**^5^ | 2,8 | 2,1 | 2,9 | 2,4 | 2,9 | 1,8 | 2,2 | 1,1 | 1,9 | 1,0 | 2,5 | 1,9 |
| **Block consumption (g/bird)** | 2,7 | 1,5 | 6,1 | 6,0 | 2,7 | 1,5 | 5,1 | 4,0 | 6,2 | 6,7 | 7,5 | 7,4 |
| **Bale consumption (g/bird)** | 171 | 139 | 415 | 487 | 461 | 554 | 358 | 508 | 495 | 575 | 750 | 1198 |

*^1^ from D0 to D32 for Ross 308, from D0 to D46 for JA 757 and from D0 to D39 for other strains, as a percentage of birds on D0; ^2^ from D11 to D32 for Ross 308, from D11 to D46 for JA 757 and from D11 to D39 for other strains, as a percentage of birds on D11; ^3^ Scored only for broilers with a feathered breast; ^4^ Interacting with enrichments means total of Perching on bales and the scale, Touching the bales and the block, Pecking at bales and the block; ^5^ mean of the 3 areas per pen, from 0 to 4, 0 being a completely dry and flaky litter.*
